# Adjuvant Chemotherapy for Muscle-invasive Bladder Cancer: A Systematic Review and Meta-analysis of Individual Participant Data from Randomised Controlled Trials

**DOI:** 10.1016/j.eururo.2021.09.028

**Published:** 2022-01

**Authors:** Advanced Bladder Cancer (ABC) Meta-analysis Collaborators Group, Sarah Burdett, David J. Fisher, Claire L. Vale, Cora N. Sternberg, Noel W. Clarke, Mahesh K.B. Parmar, Aldo V. Bono, Francesco Cognetti, Laurence Collette, Richard J. Cote, Peter J. Goebell, Susan Groshen, Jan Lehmann, Alexander I. Rolevich, Roland W. Sonntag, Michael Stockle, Urs E. Studer, Frank M. Torti, Alexander G. Zhegalik, Jayne F. Tierney

**Affiliations:** a MRC Clinical Trials Unit at UCL, London, UK; b Weill Cornell Medicine, New York Presbyterian Hospital, New York, NY, USA; c The Christie and Salford Royal NHS Foundation Trusts, Manchester, UK; d Circolo Hospital and Macchi Foundation, Varese, Italy; e Sapienza University of Rome, Rome, Italy; f European Organisation for Research and Treatment of Cancer, Brussels, Belgium; g Saint Louis University, St. Louis, MO, USA; h Erlangen University Hospital, Erlangen, Germany; i University of Southern California, Los Angeles, CA, USA; j Krankenhuis Kiel, Kiel, Germany; k Alexandrov National Research Cancer Center, Minsk, Belarus; l University of Bern, Bern, Switzerland; m Saarland University, Saarbrücken, Germany; n University of Connecticut, UConn, CT, USA

**Keywords:** Bladder cancer, Chemotherapy, Individual participant data, Meta-analysis, Systematic review

## Abstract

**Context:**

Our prior systematic review and meta-analysis of individual participant data (IPD) suggesting a benefit of adjuvant chemotherapy for muscle-invasive bladder cancer was limited by the number and size of included randomised trials. We have updated results to include additional trials, providing the most up-to-date and reliable evidence of the effects of this treatment.

**Objective:**

To investigate the role of adjuvant cisplatin-based chemotherapy in the treatment of muscle-invasive bladder cancer.

**Evidence acquisition:**

Published and unpublished trials were sought via searches of bibliographic databases, trials registers, conference proceedings, and hand searching. Updated IPD were centrally collected, checked, and analysed. Results from individual randomised controlled trials (RCTs) were combined using a two-stage fixed-effect model. Prespecified analyses explored any variation in effect by trial and participant characteristics.

**Evidence synthesis:**

Analyses of ten RCTs (1183 participants) demonstrated a benefit of cisplatin-based adjuvant chemotherapy on overall survival (hazard ratio [HR] = 0.82, 95% confidence interval [CI] = 0.70–0.96, *p* = 0.02). This represents an absolute improvement in survival of 6% at 5 yr, from 50% to 56%, and a 9% absolute benefit when adjusted for age, sex, pT stage, and pN category (HR = 0.77, 95% CI = 0.65–0.92, *p* = 0.004). There was no clear evidence that the effect varied by trial or participant characteristics. Adjuvant chemotherapy was also shown to improve recurrence-free survival (HR = 0.71, 95% CI = 0.60–0.83, *p* < 0.001), locoregional recurrence-free survival (HR = 0.68, 95% CI = 0.55–0.85, *p* < 0.001), and metastasis-free survival (HR = 0.79, 95% CI = 0.65–0.95, *p* = 0.01), with absolute benefits of 11%, 11%, and 8%, respectively.

**Conclusions:**

This systematic review and meta-analysis demonstrates that cisplatin-based adjuvant chemotherapy is a valid option for improving outcomes for muscle-invasive bladder cancer.

**Patient summary:**

We looked at the effect of cisplatin-based chemotherapy on outcomes in participants with muscle-invasive bladder cancer. We gathered this information from eligible randomised controlled trials. We demonstrated that cisplatin-based chemotherapy is a valid option for improving outcomes of muscle-invasive bladder cancer.

## Take Home Message

This systematic review and meta-analysis, based on updated individual participant data, demonstrates that cisplatin-based adjuvant chemotherapy is a valid option for improving outcomes for muscle-invasive bladder cancer.

## 1 Introduction

Our prior systematic review and meta-analysis of individual participant data (IPD) suggested a benefit of adjuvant chemotherapy for muscle-invasive bladder cancer, but was limited by the small number of trials and patients [1]. As around 30% of patients present with muscle-invasive disease, half of whom are likely to have occult metastases, these promising results highlighted the need to continue evaluating adjuvant chemotherapy in on-going randomised trials.

Since the original adjuvant chemotherapy meta-analysis of IPD, five additional relevant randomised controlled trials (RCTs) [2], [3], [4], [5], [6] have been completed and reported. Therefore, we have updated the systematic review and IPD meta-analysis in order to provide the most up-to-date and reliable evidence of the effects of adjuvant cisplatin-based chemotherapy in muscle-invasive bladder cancer.

## 2 Evidence acquisition

### 2.1 Methods

We aimed to investigate the role of adjuvant cisplatin-based chemotherapy in the treatment of muscle-invasive bladder cancer, including whether any predefined participant subgroups benefit more (or less) from this treatment. Unless otherwise stated, all methods were prespecified in a protocol, which was registered in PROSPERO in November 2017 (RecordID = 79637).

### 2.2 Trial eligibility

#### 2.2.1 Study type

Trials were eligible if these were randomised properly; initiated after January 1, 1965; and closed to accrual.

#### 2.2.2 Treatment comparisons

RCTs were eligible if these aimed to compare adjuvant cisplatin-based chemotherapy plus local treatment versus (1) the same local treatment alone or (2) the same local treatment and then adjuvant cisplatin-based chemotherapy on recurrence. Local treatment could include radical cystectomy, preoperative radiotherapy + cystectomy, or radical radiotherapy ± postradiotherapy salvage cystectomy for local failure, and must have been the same on both arms. Trials should be unconfounded by the use of additional treatments.

#### 2.2.3 Participants

Trials should have aimed to include participants with biopsy-proven transitional cell carcinoma with muscle-invasive bladder cancer who had not received neoadjuvant chemotherapy.

### 2.3 Trial identification

Published and unpublished trials were sought, with no language restrictions, using RCT search filters for Medline and Embase [7] with additional MeSH and free-text terms for bladder cancer and chemotherapy. These were supplemented by searching trial registers (Cochrane Collaboration CENTRAL database, ClinicalTrials.gov), conference proceedings (American Society of Clinical Oncology [ASCO; 2004–2020], ASCO GU [2008–2020], European Cancer Organisation/European Society for Medical Oncology [2004–2020], American Urological Association/European Association of Urology [2004–2020], plus review articles and reference lists of trial publications (Supplementary material). Collaborators were asked whether they knew of any additional trials. We searched for eligible trials published since 2005, the year of the previous systematic review. Searches were first carried out in 2017 and updated until August 2020.

### 2.4 Data collection and checking

As in the 2005 review, for all new eligible trials and all randomised participants, data were sought on the date of randomisation; treatment allocation; local treatment; age; sex; pathological T, N, and M stage; grade; performance status; local recurrence/progression; distant recurrence; any recurrence; treatment on recurrence; survival; cause of death; and event or last follow-up based on a pre-prepared data dictionary. Variables were recoded or derived as necessary, in order to standardise across trials.

Standard methods were used to check for missing data and the validity, range, and consistency of variables [8]. The cumulative patterns of treatment allocation, allocation by days of the week, and balance of baseline characteristics by treatment group were used to check the integrity of the randomisation process. In addition, the follow-up of surviving participants was checked to ensure that it was up to date and balanced by arm. Inconsistencies were resolved, up-to-date follow-up were obtained (where possible), and the final dataset was verified by the relevant contact for each trial. Results of these checks, together with information on the design and conduct of the included trials, were used to make risk of bias assessments for the randomisation process, deviations from the intended interventions, missing outcome data, and measurement of the outcome for each trial. Note that we used the revised risk of bias tool [9] rather than the original one specified in the protocol.

The new trial data were combined with the IPD obtained for the 2005 review for this update, which had been processed similarly.

### 2.5 Analysis

#### 2.5.1 Definition of outcomes

The primary outcome, overall survival, was defined as the time from randomisation until death from any cause, with living participants or those lost to follow-up censored on the date of last follow-up. Secondary outcomes were locoregional recurrence-free survival, metastasis-free survival, and overall recurrence-free survival. Locoregional recurrence-free survival was defined as the time from randomisation until locoregional recurrence (as the first recurrence event) or death. In individual trials, locoregional recurrence was defined as a recurrence within the pelvis, and affected nodes were classed as metastases. Metastasis-free survival was defined as the time from randomisation until distant metastases (as the first recurrence event) or death. Not all trials collected data on subsequent events, and for those that did, we cannot be sure that they were collected in a systematic and consistent way. Therefore, to avoid the potential bias from under-reporting of subsequent events, for trials that recorded only the first recurrence, participants having a locoregional recurrence were censored in the analysis of metastases, and vice versa. For participants with locoregional recurrence and metastasis recorded on different dates, only the earlier event was analysed fully, with the subsequent event censored on the date of the first. Participants with a locoregional recurrence and a metastasis recorded on the same date were counted in both analyses. All other participants without recurrence were censored on the date of death or last follow-up.

Overall recurrence-free survival was defined as the time from randomisation to first recurrence (whether local or metastases) or death; those alive without recurrence were censored as described above, or on the date of last follow-up.

#### 2.5.2 Analysis of main effects

Unless otherwise stated, all analyses were prespecified in the protocol and carried out on an intention-to-treat basis.

Cox regression models on censored time-to-event data were used to produce hazard ratio (HR) estimates of the effect of adjuvant treatment versus control. HRs were calculated for each trial individually and then combined across all trials using the fixed-effect inverse-variance meta-analysis model [10].

The DerSimonian and Laird [11] random-effects model was used to assess the robustness of the results. Chi-square heterogeneity tests were used to assess across-trial differences (heterogeneity) in the effect of treatment or in treatment-by-covariate interactions. Results are also presented as nonstratified Kaplan-Meier curves [12]. The median follow-up was computed for all participants using the reverse Kaplan-Meier method [13].

#### 2.5.3 Analyses by trial characteristics

To explore the potential impact of trial-level characteristics on the effect of adjuvant chemotherapy, analyses were prespecified by planned chemotherapy regimen (single agent cisplatin or cisplatin combination) and planned (control arm) treatment on relapse (no further treatment or cisplatin chemotherapy). Pooled HRs were calculated for each prespecified trial subgroup, with differences in treatment effect across trial subgroups assessed using chi-square tests.

#### 2.5.4 Analyses by participant characteristics

To explore any impact of participant characteristics on the effect of adjuvant chemotherapy, analyses were prespecified by age, sex, performance status, pT category, pN category, and grade. The relevant treatment-by-covariate interaction term was included in a Cox regression model for each trial. The resulting within-trial interactions (HRs) were then pooled across trials using the stratified-by-trial, fixed-effect model [14]. These analyses were carried out for each covariate in turn and were focused on the primary outcome of overall survival. If insufficient data were available, categories were combined or analyses were not conducted.

Absolute differences in outcome at 5 yr were calculated from the relevant HR and the control group baseline event rate [15]. All *p* values are two sided.

#### 2.5.5 Exploratory analyses

In addition to the planned analyses described, we conducted exploratory analyses to assess whether the modest imbalances in participant characteristics by treatment arm observed for some included trials might impact the results of the trials and the meta-analysis. Covariate adjustment is recommended in the context of randomised trials as it can increase power as well as correcting for chance imbalances [16]. In the meta-analysis context, an additional advantage is that estimates are made more comparable across trials [17]. The Cox model for treatment effect in each trial was adjusted for the following covariates: age (continuous), sex, pT stage, and pN stage. As these covariates were not available for all participants, we also conducted an unadjusted Cox regression analysis for the subset of participants with complete covariate data so that we could check whether missing cases had any impact on the results. We carried out similar analyses of overall recurrence-free survival.

We also carried out exploratory analyses of the effects of adjuvant chemotherapy on survival by the pT and pN stage subgroup categories used in the analysis of the largest included trial [5]. As the planned analysis by pT stage is confounded by pN stage and vice versa, and therefore difficult to interpret and potentially misleading, we also carried out an exploratory analysis by pathological stage [18].

## 3 Evidence synthesis

### 3.1 Trials and data obtained

We identified 13 eligible RCTs (1447 participants; Supplementary Fig. 1); 12 were published in full or as an abstract [2], [3], [4], [5], [6], [19], [20], [21], [22], [23], [24], [25], [26] and one trial (by Omura et al) was unpublished. We found no on-going trials. We already held IPD for six of these trials (491 participants) included in the 2005 analysis, and we obtained IPD for four new trials (692 participants). Data could not be obtained for three trials (264 participants), Omura et al [2], [26]. Therefore, this meta-analysis is based on data from ten trials (1183 participants) [3], [4], [5], [6], [19], [20], [21], [22], [23], [24], [25], representing 82% of randomised participants from all known eligible trials and more than double the amount of participants than in 2005.

### 3.2 Characteristics of included trials

Included trials were conducted between 1980 and 2014, and accrued between 49 and 284 participants (Table 1). The median follow-up across all trials was 6.0 yr (3.9–14.8 yr), and all trials planned cystectomy as local treatment. Six trials (47% participants) [3], [19], [20], [21], [23], [24], [25] randomised participants to local treatment plus adjuvant cisplatin-based chemotherapy versus local treatment alone. Four trials (53% participants) [4], [5], [6], [22] randomised participants to local treatment plus adjuvant cisplatin-based chemotherapy versus local treatment plus deferred adjuvant cisplatin-based chemotherapy on recurrence. One of these trials [4] had second randomisation to the timing of cisplatin (day 2 or 15). One trial [21] used the single agent cisplatin, and four trials [3], [5], [23], [24], [25] used cisplatin in combination with methotrexate, vinblastine, and either doxorubicin or epirubicin, one trial [19] used it in combination with cyclophosphamide and doxorubicin, one trial [20] used it in combination with methotrexate, one trial [22] used it in combination with methotrexate and vinblastine, and three trials [4], [5], [6] used it in combination with gemcitabine. One trial [6] planned two cycles of chemotherapy, four trials [3], [21], [23], [24], [25] planned three cycles, and five trials [4], [5], [19], [20], [22] planned four cycles; the planned cisplatin dose ranged from 70 to100 mg/m^2^, giving a total cisplatin dose of between 150 and 400 mg/m^2^ (Table 1). Although data on subsequent therapies were requested, very little were received. Only two of the six trials, which did not specify treatment on recurrence in the protocol, provided these data. This amounted to 36 patients in total.

**Table 1:** Characteristics of included trials

Trial	Accrual years	Number of participants	Stage	Treatment details	Control treatment details	Reason for trial stopping early	Median follow-up (yr)
*Trial randomised between local treatment plus adjuvant chemotherapy or local treatment alone*							
Skinner [19]	1980–1988	102	pT3-pT4, pN+, M0	Cystectomy + 4 cycles of CAP: Cisplatin 100 mg/m^2^ Cyclophosphamide 600 mg/m^2^ Doxorubicin 60 mg/m^2^	Cystectomy	Benefit of treatment seen in trial	14.5
Bono [20]	1984–1987	90 a	pT2-pT4a, pN0, M0	Cystectomy + 4 cycles of: Cisplatin 70 mg/m^2^ Methotrexate 40 mg/m^2^	Cystectomy	Did not stop early	3.4
Studer [21]	1984–1989	91	pT1 (grade 2)-pT4, pN1–2, M0	Cystectomy + 3 cycles of cisplatin 90 mg/m^2^	Cystectomy	Smaller difference than expected seen between treatments at interim analysis	6.4
Stöckle [23], Lehmann [24]	1987–1990	49	pT3b-pT4a, pN+, M0	Cystectomy + 3 cycles of MVEC or MVAC: Cisplatin 70 mg/m^2^ Methotrexate 30 mg/m^2^ Vinblastine 3 mg/m^2^ Adriamycin 30 mg/m^2^ Epirubicin 45 mg/m^2^	Cystectomy	Benefit of treatment seen in trial at interim analysis	14.8
Otto [25]	1993–1999	108	pT3, N1–2, M0	Cystectomy + 3 cycles of MVEC: Cisplatin 70 mg/m^2^ Methotrexate 30 mg/m^2^ Vinblastine 3 mg/m^2^ Epirubicin 45 mg/m^2^	Cystectomy	Did not stop early	3.9
Stadler [3]	1997–2006	114	pT1-pT2, pN0, M0 (all p53+)	Cystectomy + 3 cycles of MVAC: Cisplatin 70 mg/m^2^ Methotrexate 30 mg/m^2^ Vinblastine 3 mg/m^2^ Doxorubicin 30 mg/m^2^	Cystectomy	Smaller difference than expected seen between treatments at interim analysis	5.4
*Trial randomised between local treatment plus adjuvant chemotherapy or local treatment plus chemotherapy on relapse*							
Freiha [22]	1986–1993	51 b	pT3b-pT4, any pN, M0	Cystectomy + 4 cycles of CMV: Cisplatin 100 mg/m^2^ Methotrexate 30 mg/m^2^ Vinblastine 4 mg/m^2^	Cystectomy + (same) chemotherapy on relapse	Smaller difference than expected seen between treatments	5.1
Cognetti [4]	2001–2007	194	pT2 (grade 3) pT3-pT4, pN0–2, M0	Cystectomy + 4 cycles of: Cisplatin 70 mg/m^2^ Gemcitabine 1000 mg/m^2^ *Cisplatin given on day 2 or day 15*	Cystectomy + (same) chemotherapy on relapse	Accrual slower than expected	4.5
Sternberg [5]	2002–2014	284	pT3-pT4 or pN1–3, M0	Cystectomy + choice of 4 cycles of either: (1) MVAC (28-d cycle), (2) high-dose MVAC (14-d cycle), or (3) GC: Cisplatin 70 mg/m^2^ Methotrexate 30 mg/m^2^ Vinblastine 3 mg/m^2^ Adriamycin 30 mg/m^2^ Gemcitabine 1000 mg/m^2^	Cystectomy + 6 cycles (same) chemotherapy on relapse	Accrual slower than expected	6.9
Zhegalik [6]	2007–2013	100	pT3-pT4 and/or pN+, M0	Cystectomy + 2 cycles of: Cisplatin 75 mg/m^2^ Gemcitabine 1000 mg/m^2^	Cystectomy + (same) chemotherapy on relapse	Did not stop early	7.3

CAP = cyclophosphamide, doxorubicin, and cisplatin; CMV = cisplatin, methotrexate, and vinblastine; GC = gemcitabine and cisplatin; MVAC = methotrexate, vinblastine, doxorubicin, and cisplatin; MVEC = methotrexate, vinblastine, epirubicin, and cisplatin.

a Ninety participants supplied and used in 2005 analysis.

b Fifty-five randomised and 51 supplied.

While there were some small imbalances in participant characteristics by treatment, based on information supplied by investigators and direct checks of the IPD, we judged the risk of bias [9] associated with the randomisation process to be low. In addition, the risk of bias associated with deviations from the intended interventions, missing outcome data, and measurement of the outcome in these ten trials was considered to be low (Supplementary Table 1).

Two trials stopped accrual early due to an observed benefit of treatment within the trial [19], [23], [24]. Three trials [3], [12], [22] stopped early due to a smaller than expected difference between treatments or futility analysis, and two [4], [5] stopped due to slower than expected accrual (Table 1).

### 3.3 Characteristics of included participants

Data on age, sex, pT category, pN category, and grade were obtained for all ten trials (Table 2). Based on the available data, participants were mostly male (84%) with a median age of 62 yr (range 23–85 yr). Their tumours were mostly pT3 (51%) or pT2 (19%), and pN0 (55%) or pN1 (22%). Data on performance status were provided for only four trials (56% participants) [4], [5], [6], [20], and based on these, the majority of participants had (WHO performance status/ECOG perfomance status) performance status 0 or 1 (90%). Data on grade were obtained, but different systems were used, and these could not be compared reliably.

**Table 2:** Characteristics of included participants

Participant characteristic	Treatment (*N* = 600)	Control (*N* = 583)
Age (yr)		
Median	61.5	62
Range	23–78	30–85
Sex, *n* (%)		
Male	512 (86)	478 (82)
Female	86 (14)	103 (18)
Unknown	2 (<1)	2 (<1)
pT stage, *n* (%)		
pT0	8 (1)	4 (<1)
pT1	33 (6)	23 (4)
pT2	115 (19)	114 (20)
pT3	305 (51)	303 (52)
pT4a	96 (16)	94 (16)
Other	9 (1)	17 (2)
Unknown	34 (6)	28 (5)
pN category, *n* (%)		
pN0	324 (54)	331 (57)
pN1	131 (22)	130 (22)
pN2	125 (21)	108 (19)
pN3	4 (<1)	0
Unknown	16 (3)	14 (2)
Performance status^a^*n* (%)		
0	223 (66)	210 (64)
1	78 (23)	85 (26)
2	3 (<1)	3 (1)
Unknown	35 (11)	31 (9)

a Performance status was available for four of the ten trials (56% of participants).

### 3.4 Treatment effects on overall survival

#### 3.4.1 Main effects

**Fig. 1:**
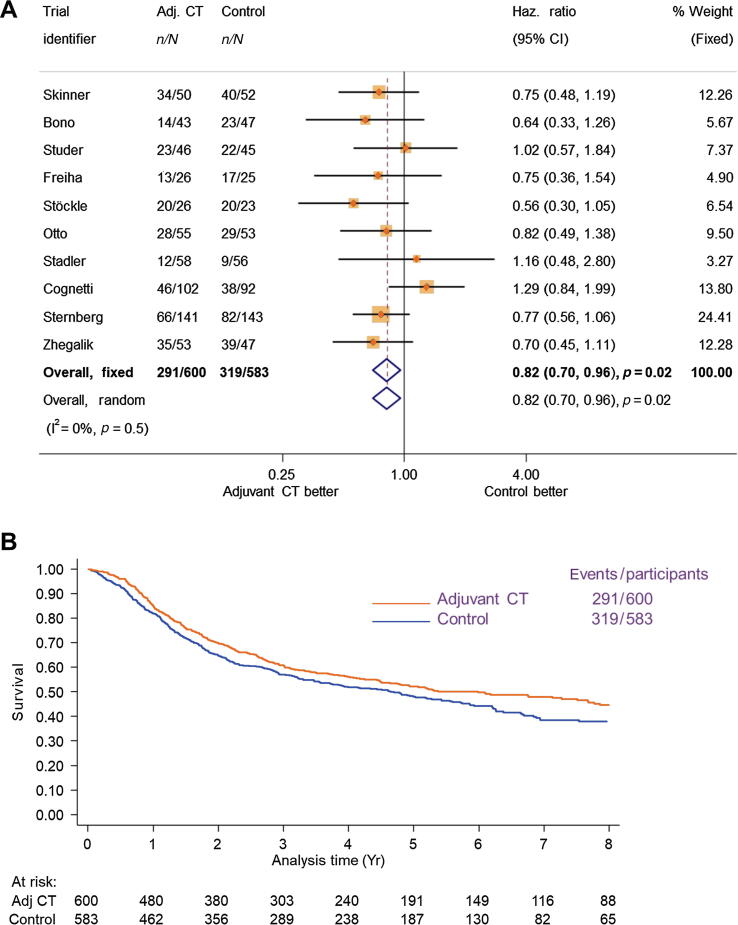
(A) Forest plot and (B) Kaplan-Meier curves (nonstratified) of the effect of adjuvant chemotherapy on overall survival. In figure (A), each trial is represented by a square, the centre of which denotes the hazard ratio for that trial (comparison), with the horizontal lines showing the 95% and 99% confidence intervals (CIs). The size of the square is directly proportional to the amount of information contributed by the trial. The black diamond gives the pooled hazard ratio from the fixed-effect model; the centre of this diamond denotes the hazard ratio and the extremities of the 95% CI. Adj CT = adjuvant chemotherapy; CI = confidence interval; Haz. Ratio = hazard ratio.

Overall survival results were based on ten RCTs (1183 participants and 610 deaths) and showed a clear benefit of adjuvant chemotherapy (HR = 0.82, 95% confidence interval [CI] = 0.70–0.96, *p* = 0.02), with no clear evidence of statistical heterogeneity (*p* = 0.05, I^2^ = 0%; Fig. 1). These translate to a 6% (95% CI = 1–11%) absolute improvement in overall survival at 5 yr (from 50% to 56%). Results were the same when a random-effects model was used (HR = 0.82, 95% CI = 0.70–0.96, *p* = 0.02).

**Fig. 2:**
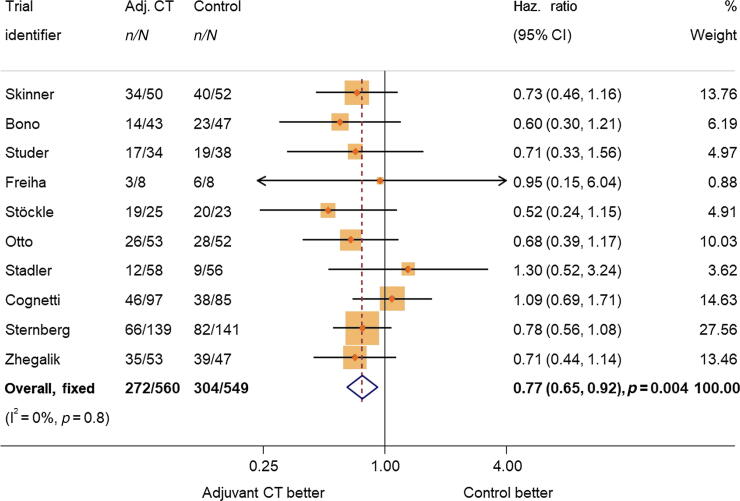
Effect on overall survival adjusted by age, sex, pT stage, and pN status. Adj CT = adjuvant chemotherapy; CI = confidence interval; Haz. Ratio = hazard ratio.

Results of an exploratory unadjusted analysis of the subset of participants (94%) with complete data on age, sex, pT stage, and pN stage were very similar to the main analysis result (HR = 0.81, 95% CI = 0.69–0.95, *p* = 0.01). When the analysis of this same group of participants was adjusted for age, sex, pT stage, and pN stage, results showed a larger benefit of adjuvant chemotherapy on overall survival (HR = 0.77, 95% CI = 0.65–0.92, *p* = 0.004; Fig. 2), which translates to a 9% (95% CI = 3–14%) absolute improvement in survival at 5 yr (from 50% to 59%).

In response to the statistical reviewer, we conducted additional exploratory analyses of age, pT stage, and pN stage, which avoid the linearity assumption, and these gave similar results. With respect to a reference category of <55 yr, there was no clear evidence of an interaction by age (interaction HR for ≥55–<65 yr = 0.96, 95% CI = 0.60–1.55; interaction HR for ≥65 yr = 0.95, 95% CI = 0.59–1.52; joint test for interaction *p* = 1.0). With respect to a reference category of pT2, there was no clear evidence of an interaction by pT stage (interaction HR for pT3 = 0.68, 95% CI = 0.39–1.16; interaction HR for pT4a = 0.58, 95% CI = 0.31–1.09; joint test for interaction *p* = 0.2). With respect to a reference category of pN0, again there was no clear evidence of an interaction by pN category (interaction HR for pN1 = 1.44, 95% CI = 0.91–2.29; interaction HR for pN2 = 1.15, 95% CI = 0.73–1.80; joint test for interaction *p* = 0.3).

#### 3.4.2 Effects by trial characteristics

There was no clear evidence that the effect of adjuvant chemotherapy on overall survival differed according to whether cisplatin was given as a single agent or as a cisplatin combination (interaction *p* = 0.5); although as only one trial used single-agent chemotherapy, this analysis is very limited. Moreover, there was no clear difference in effect according to whether adjuvant chemotherapy was planned to be given on recurrence or not (interaction *p* = 0.6; Supplementary Figs. 2 and 3).

#### 3.4.3 Effects by participant characteristics

**Fig. 3:**
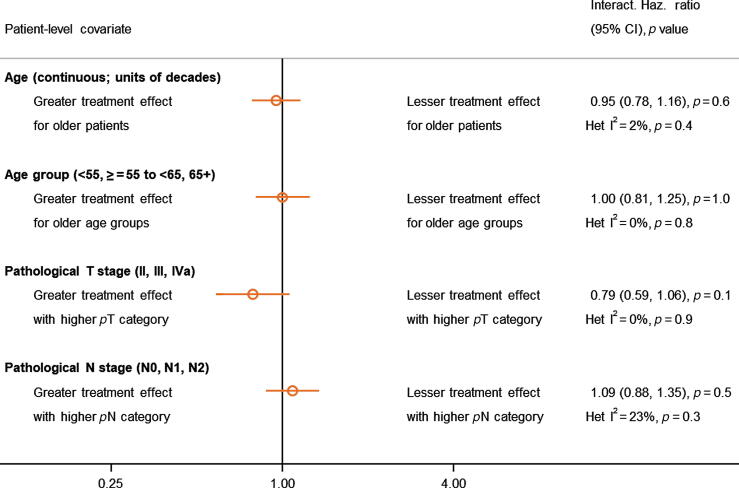
Interactions between the effect of adjuvant chemotherapy on overall survival and age, pT, and pN. The open circles represent (fixed-effect) meta-analyses of the HRs representing the interactions between the effect of chemotherapy and participant characteristics, with the horizontal line showing the 95% CI. CI = confidence interval; HR = hazard ratio; Haz. Ratio = hazard ratio; Het: Heterogeneity.

We found no clear evidence that the effect of adjuvant chemotherapy on overall survival differed by age (continuous; interaction HR = 0.95, 95% CI = 0.78–1.16, *p* = 0.6, I^2^ = 2%, Het *p* = 0.4), age (categorical; interaction HR = 1.00, 95% CI = 0.81–1.25, *p* = 1.0, I^2^ = 0%, Het *p* = 0.8), pT category (interaction HR = 0.79, 95% CI = 0.59–1.06, *p* = 0.1, I^2^ = 0%, Het *p* = 0.9), or pN category (interaction HR = 1.09, 95% CI = 0.88–1.35, *p* = 0.5, I^2^ = 23%, Het *p* = 0.3; Fig. 3, and Supplementary Figs. 4 and 5). Applying the overall HR of 0.82 translates to 5-yr survival benefits of 5% for pT2, 7% for pT3, and 7% for pT4a, and of 6% for N0, 7% for N1, and 7% for N2 at 5 yr. Prespecified analyses by sex, grade, or performance status were not carried out due to the majority of participants being in one category and/or insufficient data being available. As grade was measured on different scales, it was not reasonable to combine these in a consistent or reliable way for analysis.

An exploratory analysis based on the pT and pN subgroups used by Sternberg et al (pT1/2, pT3, pT4a; N−, N+) [5] showed no clear evidence that the effect of adjuvant chemotherapy varied by T stage (interaction HR = 0.78, 95% CI = 0.58–1.03, *p* = 0.08, I^2^ = 0%, Het *p* = 0.9) or N stage (interaction HR = 1.38, 95% CI = 0.94–2.03, *p* = 0.5, I^2^ = 31%, Het *p* = 0.2).

As the majority of participants (85%) were at American Joint Committee on Cancer (AJCC) stage IIIa and IIIb, the exploratory analyses of the effects of adjuvant chemotherapy on survival by AJCC stage were necessarily limited to these stages, and were based on six trials and 785 participants. We found no clear evidence that the effect of adjuvant chemotherapy differed according to whether participants had a stage IIIa or IIIb disease (HR = 0.95, 95% CI = 0.64–1.39, *p* = 0.8; Supplementary Fig. 6). Applying the overall HR of 0.82 translates to 5-yr survival benefits of 7% for both stage IIIa and stage IIIb.

### 3.5 Effects on recurrence-free survival

**Fig. 4:**
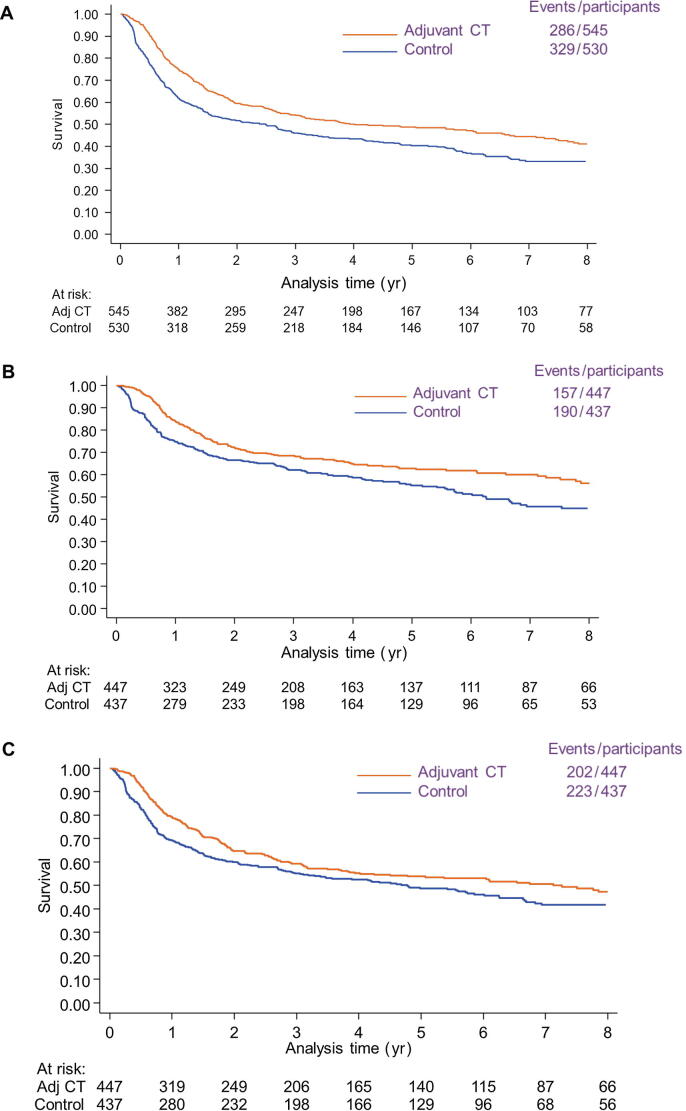
Kaplan-Meier curves (nonstratified) of the effect of adjuvant chemotherapy on (A) recurrence-free survival, (B) local recurrence-free survival, and (C) metastasis-free survival. Adj CT = adjuvant chemotherapy.

Recurrence-free survival results were based on nine RCTs (1075 participants) and included 615 events, which comprised 472 recurrences and 143 deaths without recurrence (Supplementary Fig. 7). These results showed a benefit of adjuvant chemotherapy (HR = 0.71, 95% CI = 0.60–0.83, *p* < 0.001), which translates to an 11% (6–16%) absolute improvement in recurrence-free survival from 50% to 61% at 5 yr. There was modest evidence of statistical heterogeneity (*p* = 0.12, I^2^ = 38%), but the results were very similar when the random-effects model was used (HR = 0.72, 95% CI = 0.58–0.89, *p* = 0.002; Fig. 4). Based on the subset of participants (94%) with complete data on age, sex, pT stage, and pN stage, results of an exploratory unadjusted analysis again were very similar to those of the primary analysis (HR = 0.71, 95% CI = 0.60–0.84, *p* < 0.001), whereas the adjusted analysis showed a larger benefit of adjuvant chemotherapy (HR = 0.67 adjusted, 95% CI = 0.56–0.80, *p* < 0.001). This translates to a 13% (95% CI = 6–18%) absolute improvement in survival at 5 yr (from 50% to 63%).

### 3.6 Effects on locoregional recurrence-free survival

Locoregional recurrence-free survival results were based on six RCTs (884 participants, 347 events) and showed a benefit of adjuvant chemotherapy (HR = 0.68, 95% CI = 0.55–0.85, *p* < 0.001). This translates to an 11% (95% CI = 5–16%) absolute improvement in locoregional recurrence-free survival from 60% to 71% at 5 yr. There was no clear evidence of statistical heterogeneity (*p* = 0.9, I^2^ = 0%; Fig. 4), and results were the same when a random-effects model was used (HR = 0.68, 95% CI = 0.55–0.85, *p* < 0.001).

### 3.7 Metastasis-free survival

Metastasis-free survival results were based on six RCTs (884 participants, 425 events) and showed a benefit of adjuvant chemotherapy (HR = 0.79, 95% CI = 0.65–0.95, *p* = 0.02). This translates to an 8% (95% CI = 2–14%) absolute improvement in metastasis-free survival from 50% to 58% at 5 yr. There was no clear evidence of statistical heterogeneity (*p* = 0.3, I^2^ = 11%; Fig. 4), and results were similar when a random-effects model was used (HR = 0.79, 95% CI = 0.64–0.97, *p* = 0.02).

### 3.8 Toxicity

We aimed to describe the toxicity on the treatment arm for included trials. However, different classification systems have been used, the data have been reported inconsistently, and further data could be provided for some trials only. However, the chemotherapy regimens used in these trials are well known, as are their toxicity profiles. No trial reported anything unexpected; haematological events, and nausea and vomiting were the most commonly reported effects.

### 3.9 Summary of results

We have shown a 6% absolute benefit of cisplatin-based adjuvant chemotherapy in 5-yr survival of participants with muscle-invasive bladder cancer, and a 9% absolute benefit when adjusted for age, sex, pT stage, and pN category. There was no clear evidence that the effect varied by trial or participant characteristics. Adjuvant chemotherapy was also shown to improve recurrence-free survival, locoregional recurrence-free survival, and metastasis-free survival, with 5-yr absolute benefits of 11%, 11%, and 8%, respectively.

### 3.10 Strengths

We have been able to include IPD from ten out of 13 eligible trials, comprising 82% of all known randomised participants, which represent more than double the amount of participants included in the 2005 analysis [1]. The collection of IPD has enabled us to include data on all planned outcomes consistently defined across all trials, and to explore the effect of treatment across participant subgroups reliably. The inclusion of updated follow-up for some of the trials [6], [19], [20], [21], [23], [24] has allowed us to provide the most up-to-date estimates of the effect of adjuvant chemotherapy, as well as alleviating the potential for bias in trials that stopped early for benefit [1]. By conducting analyses of overall and recurrence-free survival adjusted by age, pT status, and pN category, we were also able to account for imbalances in baseline characteristics in some of the included trials.

### 3.11 Limitations

Almost one-third of the participants in these trials did not receive all the chemotherapy cycles as planned; some trials administered fewer chemotherapy cycles and lower total doses of cisplatin than others (Table 1). This is what we might expect in clinical practice, but it may have served to dilute the estimated overall effect of adjuvant chemotherapy and makes it difficult to recommend an optimum number of cycles of chemotherapy.

Trials that terminate early for a benefit or harm observed within the trial are more prone to bias [27]. Two included trials stopped accrual early due to an observed benefit of treatment within the trial [19], [23], [24]. This was discussed in the original meta-analysis, and updated follow-up was obtained for both of these, which seemed to mitigate the potential bias [1]. Three included trials [3], [21], [22] stopped early due to a smaller than expected difference between treatments or futility analysis, and two [4], [5] stopped due to slower than expected accrual, which is unlikely to have introduced bias (Table 1).

For the original IPD meta-analysis, data were not available for two eligible trials (Omura et al, unpublished) [26], and data for a further trial [2] were not available for this update, despite repeated requests to the investigator. One of these trials was never published (Omura et al, unpublished), and another did not publish survival data [26]. Combination of the HR for the other trial (presented in 2010 [2]) with our IPD results in a sensitivity analysis gives an overall HR of 0.77 (0.67, 0.90; *p* = 0.003, Het *p* = 0.4), which is in keeping with the IPD results.

### 3.12 Context

Results of Sternberg et al [5] included here suggest a difference in the effect of adjuvant chemotherapy on survival by nodal status, with a greater effect in those without involved nodes. However, there was no differential effect by nodal status in the Sternberg et al’s [5] analysis of progression-free survival or the meta-analysis results presented here. Moreover, these analyses do not take into account the accompanying pT stage, making interpretation challenging, whereas our exploratory analysis by p stage [18] does and fails to provide clear evidence that the effect of adjuvant chemotherapy on survival is modified by pathological stage.

### 3.13 Implications

Neoadjuvant chemotherapy is currently recommended [28] to treat most patients with muscle-invasive bladder cancer, and our prior neoadjuvant chemotherapy systematic review and meta-analysis based on IPD [29] showed a 5% absolute improvement in survival. Outcomes may be improved further if results from on-going trials show benefits of immunotherapy and if prognostic biomarkers can be better defined. With adjuvant chemotherapy, there is a similar absolute survival benefit at 5 yr (6%) and a reduction in local and distant failure rates. With no real data to compare the two treatments, we cannot say whether one is better for either all patients or specific types of patients. However, the meta-analysis shows that there is now a choice between the two treatments, depending on circumstances and clinician and patient preference. Consideration of immunotherapy in these patients is further in the future, as current research is focused on individuals who have received neoadjuvant chemotherapy or those who are unsuitable for cisplatin-based chemotherapy.

## 4 Conclusions

This systematic review and meta-analysis, based on updated IPD, demonstrates that cisplatin-based adjuvant chemotherapy is a valid option for improving outcomes for muscle-invasive bladder cancer.

  ***Author contributions***: Sarah Burdett had full access to all the data in the study and takes responsibility for the integrity of the data and the accuracy of the data analysis.

*Study concept and design*: Burdett, Tierney, Vale, Clarke, Parmar, Sternberg.

*Acquisition of data*: Burdett, Vale.

*Analysis and interpretation of data*: Burdett, Fisher, Tierney, Vale.

*Drafting of the manuscript*: Burdett, Fisher, Tierney, Vale.

*Critical revision of the manuscript for important intellectual content*: Burdett, Fisher, Tierney, Vale, Clarke, Cognetti, Collette, Cote, Goebell, Groshen, Lehmann, Parmar, Rolevich, Sternberg, Stöckle, Studer, Torti, Zhegalik.

*Statistical analysis*: Fisher.

*Obtaining funding*: None.

*Administrative, technical, or material support*: Burdett.

*Supervision*: Clarke, Parmar, Sternberg.

*Other*: Provision of IPD: Bono, Cognetti, Collette, Cote, Goebell, Groshen, Lehmann, Rolevich, Stöckle, Sonntag, Sternberg, Studer, Torti, Zhegalik.

  ***Financial disclosures***: Sarah Burdett certifies that all conflicts of interest, including specific financial interests and relationships and affiliations relevant to the subject matter or materials discussed in the manuscript (eg, employment/affiliation, grants or funding, consultancies, honoraria, stock ownership or options, expert testimony, royalties, or patents filed, received, or pending), are the following: Dr. Sternberg reports consultancy fees from Pfizer, MSD, Merck, AstraZeneca, Astellas, Sanofi-Genzyme, Roche-Genentech, Incyte, BMS, Foundation Medicine, Immunomedics Now, Gilead, Medscape, UroToday, CCO Clinical, Janssen, and NCI, outside the submitted work. Professor Cognetti reports personal fees from GlaxoSmithKline, Roche, Novartis, from Amgen, Pfizer, AstraZeneca, MSD (Merck Sharp & Dohme), Bristol Meyers Squibb, Astellas Oncology, Eli Lilly, and Genomic Health, outside the submitted work. Dr. Goebell reports to have received honoraria/support as a speaker from Astellas, AstraZeneca, Bayer, BMS, Eisai, EUSA, Ipsen, Janssen, Novartis, Pfizer, Roche, and Sanofi; and has received honoraria for participation in expert rounds from Astellas, AstraZeneca, Bayer, BMS, Eisai, EUSA, Ipsen, Janssen, Novartis, Pfizer, Roche, and Sanofi, outside the submitted work. Dr. Rolevich reports personal fees and nonfinancial support from Pfizer, and personal fees from Astellas Pharma and Sanofi-Aventis, outside the submitted work. Dr. Zhegalik reports personal fees from Astellas Pharma, outside the submitted work. All other authors have nothing to declare.

  ***Funding/Support and role of the sponsor***: The Project Management Group was funded by the UK Medical Research Council (MC_UU_12023/25). The sponsors of the study had no role in study design, data collection, data analysis, data interpretation, or writing of the report. The corresponding author had full access to all the data in the study and had final responsibility for the decision to submit for publication.

  ***Acknowledgements***: The Advanced Bladder Cancer (ABC) Meta-analysis Collaborators Group thanks all participants who took part in the trials and contributed to this research. The meta-analysis would not have been possible without their participation or without the collaborating institutions that provided their trial data. The Project Management Group was funded by the UK Medical Research Council (MC_UU_12023/25). We also thank Diana Giannarelli, Regina Elena National Cancer Institute, Rome, Italy, for her help with the processing of data for the Cognetti et al trial.

  **Advanced Bladder Cancer (ABC) Meta-analysis Collaborators Group**:

***Project Management Group:***

S. Burdett, MRC Clinical Trials Unit at UCL, London, UK

D.J. Fisher, MRC Clinical Trials Unit at UCL, London, UK

C.L. Vale, MRC Clinical Trials Unit at UCL, London, UK

J.F. Tierney, MRC Clinical Trials Unit at UCL, London, UK

***International Advisory Group:***

N.W. Clarke, The Christie and Salford Royal NHS Foundation Trusts, Manchester, UK

M.K.B. Parmar, MRC Clinical Trials Unit at UCL, London, UK

C.N. Sternberg, Englander Institute for Precision Medicine, Weill Cornell Medicine New York-Presbyterian, NY, USA

***Collaborators who supplied IPD:***

*Stockle trial:*

M. Stöckle, Saarland University; Homburg/Saar, Germany

J. Lehmann, Städtisches Krankenhaus, Kiel, Germany

*Studer trial:*

U.E. Studer, University of Bern, Bern, Switzerland

*R.W. Sonntag (deceased), University of Bern, Bern, Switzerland*

*Frieha trial:*

F.M. Torti, University of Connecticut School of Medicine, CT, USA

*Skinner trial:*

S. Groshen (Retired), University of Southern California, Los Angeles, CA, USA

*Bono trial* ***:***

*A.V. Bono (deceased), Circolo Hospital and Macchi Foundation, Varese, Italy*

*Otto trial:*

P.J. Goebell, University Hospital Erlangen, Friedrich-Alexander Universität Erlangen-Nürnberg, Erlangen, Germany

*Cognetti trial:*

F. Cognetti, Sapienza University of Rome, Rome, Italy

*Stadler trial:*

R.J. Cote, St. Louis University School of Medicine, MO, USA

S. Groshen (Retired), University of Southern California, Los Angeles, CA, USA

*Sternberg trial:*

L. Collette, EORTC AISBL/IVZW, Brussels, Belgium

C.N. Sternberg, Englander Institute for Precision Medicine, Weill Cornell Medicine and

New York-Presbyterian, NY, USA

*Zhegalik trial:*

A.I. Rolevich Alexandrov National Research Cancer Center, Minsk, Belarus

A.G. Zhegalik, Alexandrov National Research Cancer Center, Minsk, Belarus
