# Supplementary material for: Adjuvant Chemotherapy for Muscle-invasive Bladder Cancer: A Systematic Review and Meta-analysis of Individual Participant Data from Randomised Controlled Trials
Source: Eur Urol. 2022 Jan;81(1):50–61. doi: 10.1016/j.eururo.2021.09.028 (PMC8708165; doi:10.1016/j.eururo.2021.09.028)
Supplement: Supplementary data 1 [file mmc1.docx]

# Supplementary Figure 1: PRISMA flow diagram of trial identification, screening, eligibility and inclusion for updated searches 2005-2020

**
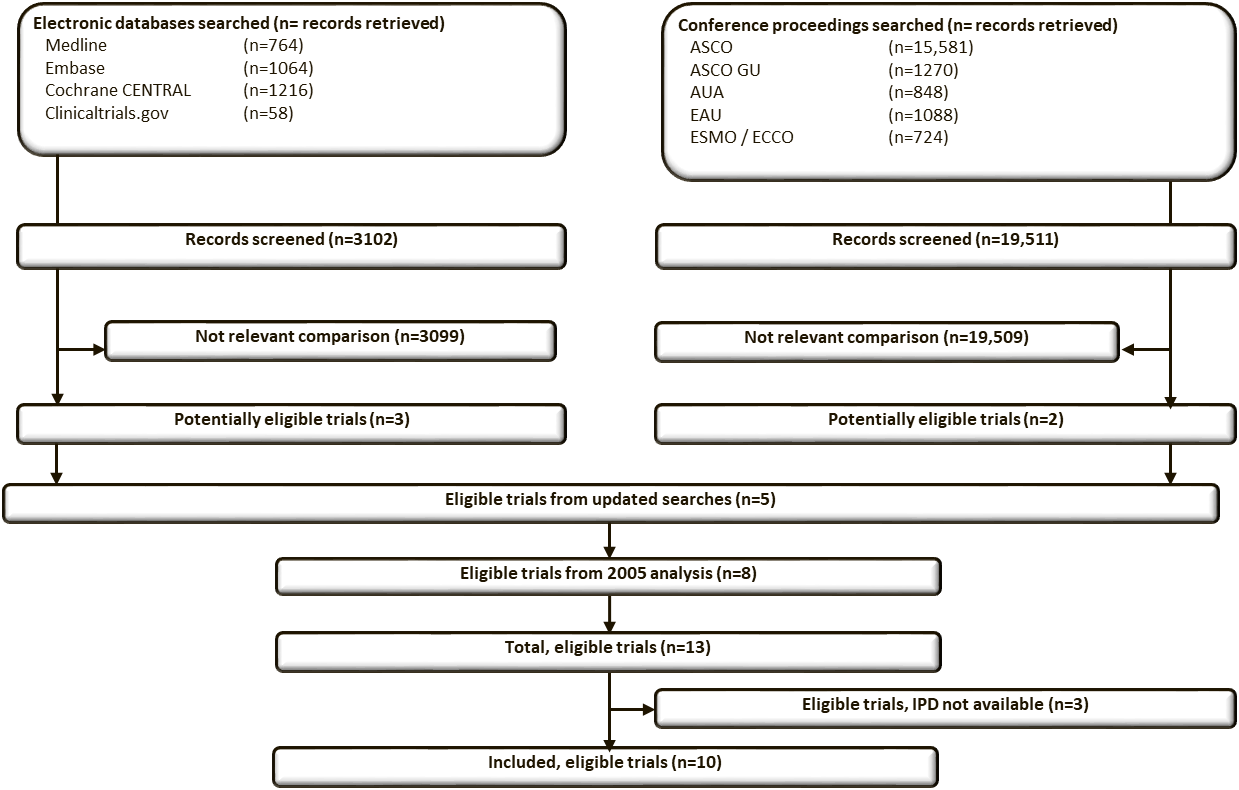
**

# Supplementary Table 1: Risk of bias assessment of included studies

|  | | | |
| --- | --- | --- | --- |
| 1. Randomisation process | 1. Deviations from the intended interventions | 1. Missing outcome data | 1. Measurement of the outcome |
| Trial – Bono | | | |
| LOW RISK  *Was allocation sequence random?*  Controlled, multicentre, centrally randomized study, balancing patients in series of 10…stratified according to the pathology report  *Was allocation sequence concealed?*  Centrally randomized  Also, IPD checks show that the pattern of allocation is balanced by treatment group over time; there were no major imbalances by group on any day of the week; and there were few weekend randomisations.  *Did baseline differences suggest a problem?*  IPD checks show some imbalances in baseline sex, pTcat, and PS | **LOW RISK**  ***Were participants aware of their assigned intervention during the trial?***  Blinding not possible in a chemotherapy versus none trial, but awareness cannot affect survival outcome.  ***Were carers and people delivering the interventions aware of participants' assigned intervention during the trial?***  Blinding not possible, but awareness would not affect how treatments were given.  ***Were there deviations from the intended intervention that arose because of the trial context?***  There were no deviations because of the context.  ***Was an appropriate analysis used to estimate the effect of assignment to intervention?*** An intention-to-treat analysis of all randomised participants was derived from the IPD. | **LOW RISK**  ***Were data available for all, or nearly all, participants randomized?***  Data on survival were provided for 90 participants, the 83 reported in the trial manuscript and an additional 7 participants excluded from the original trial analysis.  ***Other outcomes***  Data on local recurrence and metastases were provided for all participants. | **LOW RISK**  ***Was method of measuring the outcome inappropriate?***  Overall survival was derived from the IPD, according to the meta-analysis protocol and SAP  ***Could measurement of the outcome have differed between intervention groups?***  Checks of the IPD revealed that follow-up of participants was balanced by treatment group.  ***Outcome assessor aware of intervention received?***  Yes, but cannot affect the overall survival outcome.  ***Other outcomes***  All secondary outcomes were derived from IPD. Knowledge of intervention cannot affect the secondary outcomes |
| Trial – Frieha | | | |
| LOW RISK  *Was allocation sequence random?*  Randomised. Simple randomisation used.  *Was allocation sequence concealed?*  Sealed envelope  Also, IPD checks on show that the pattern of allocation is generally balanced by treatment group over time. There were no major imbalances by group on any day of the week and there were few weekend randomisations.  *Did baseline differences suggest a problem?*  IPD checks show some imbalances by baseline pTcat and grade | **LOW RISK**  ***Were participants aware of their assigned intervention during the trial?***  Blinding not possible in a chemotherapy versus none trial, but awareness cannot affect survival outcome.  ***Were carers and people delivering the interventions aware of participants' assigned intervention during the trial?***  Blinding not possible, but awareness would not affect how treatments were given.  ***Were there deviations from the intended intervention that arose because of the trial context?***  There were no deviations because of the context.  ***Was an appropriate analysis used to estimate the effect of assignment to intervention?*** An intention-to-treat analysis of all randomised participants was derived from the IPD. | **LOW RISK**  ***Were data available for all, or nearly all, participants randomized?***  Data on survival were provided for 51/55 randomised participants, including 1 participant not in the reported analysis.  ***Other outcomes***  Data on overall recurrence were provided for all participants, data were not provided for local recurrence or metastases | **LOW RISK**  ***Was method of measuring the outcome inappropriate?***  Overall survival was derived from the IPD according to the meta-analysis protocol and SAP  ***Could measurement of the outcome have differed between intervention groups?***  Checks of the IPD revealed that follow-up of participants was balanced by treatment group.  ***Outcome assessor aware of intervention received?***  Yes, but cannot affect the overall survival outcome.  ***Other outcomes***  Data were not supplied for local recurrence or metastases, RFS was derived from IPD. Knowledge of intervention cannot affect the secondary outcomes |
| Trial – Skinner | | | |
| LOW RISK  *Was allocation sequence random?*  Randomised. Minimisation procedure was used to balance the treatment groups with respect to sex, pstage and histology.  *Was allocation sequence concealed?*  Central telephone  Also, IPD checks show that the pattern of allocation is generally balanced by treatment group over time; there were no major imbalances by group on any day of the week; and there were few weekend randomisations.  *Did baseline differences suggest a problem?*  IPD checks show baseline characteristics are well-balanced. | **LOW RISK**  ***Were participants aware of their assigned intervention during the trial?***  Blinding not possible in a chemotherapy versus none trial, but awareness cannot affect survival outcome.  ***Were carers and people delivering the interventions aware of participants' assigned intervention during the trial?***  Blinding not possible, but awareness would not affect how treatments were given.  ***Were there deviations from the intended intervention that arose because of the trial context?***  There were no deviations because of the context.  ***Was an appropriate analysis used to estimate the effect of assignment to intervention?*** An intention-to-treat analysis of all randomised participants was derived from the IPD. | **LOW RISK**  ***Were data available for all, or nearly all, participants randomized?***  Data were provided for 102 participants, including 11 participants not in the reported analyses.  ***Other outcomes***  Local recurrence and metastases data were provided for all patients randomised | **LOW RISK**  ***Was method of measuring the outcome inappropriate?***  Overall survival was derived from the IPD according to the meta-analysis protocol and SAP  ***Could measurement of the outcome have differed between intervention groups?***  Checks of the IPD revealed that follow-up of participants was balanced by treatment group  ***Outcome assessor aware of intervention received?***  Yes, but cannot affect the overall survival outcome.  ***Other outcomes***  All secondary outcomes were derived from IPD. Knowledge of intervention cannot affect the secondary outcomes |
| Trial – Stockle | | | |
| LOW RISK  *Was allocation sequence random?*  Randomised, permuted blocks  *Was allocation sequence concealed?*  Central randomization list  Also, IPD checks show that the pattern of allocation is generally balanced by treatment group and over time; there were no major imbalances by group on any day of the week; and there were no weekend randomisations.  *Did baseline differences suggest a problem?*  IPD checks show some imbalances by baseline pTcat and grade | **LOW RISK**  ***Were participants aware of their assigned intervention during the trial?***  Blinding not possible in a chemotherapy versus none trial, but awareness cannot affect survival outcome.  ***Were carers and people delivering the interventions aware of participants' assigned intervention during the trial?***  Blinding not possible, but awareness would not affect how treatments were given.  ***Were there deviations from the intended intervention that arose because of the trial context?***  There were no deviations because of the context.  ***Was an appropriate analysis used to estimate the effect of assignment to intervention?*** An intention-to-treat analysis of all randomised participants was derived from the IPD. | **LOW RISK**  ***Were data available for all, or nearly all, participants randomized?***  Data were provided for all randomised participants  ***Other outcomes***  Data on overall recurrence were provided for all participants randomised, data were not provided on local recurrence or metastases | **LOW RISK**  ***Was method of measuring the outcome inappropriate?***  Overall survival was derived from the IPD according to the meta-analysis protocol and SAP.  ***Could measurement of the outcome have differed between intervention groups?***  Checks of the IPD revealed that follow-up of participants was balanced by treatment group.  ***Outcome assessor aware of intervention received?***  This cannot affect the overall survival outcome.  ***Other outcomes***  Data were not supplied for local recurrence or metastases, RFS was derived from IPD. Knowledge of intervention cannot affect the secondary outcomes |
| Trial – Studer | | | |
| LOW RISK  *Was allocation sequence random?*  Randomised. Stratified by postoperative nodal status and institution.  *Was allocation sequence concealed?*  Centrally randomised  Also, IPD checks show that the pattern of allocation is balanced by treatment group over time; there were no major imbalances by group on any day of the week; and there were no weekend randomisations.  *Did baseline differences suggest a problem?*  IPD checks show some imbalances by baseline sex, pTcat and grade | **LOW RISK**  ***Were participants aware of their assigned intervention during the trial?***  Blinding not possible in a chemotherapy versus none trial, but awareness cannot affect survival outcome.  ***Were carers and people delivering the interventions aware of participants' assigned intervention during the trial?***  Blinding not possible, but awareness would not affect how treatments were given.  ***Were there deviations from the intended intervention that arose because of the trial context?***  There were no deviations because of the context.  ***Was an appropriate analysis used to estimate the effect of assignment to intervention?***  An intention-to-treat analysis of all randomised participants was derived from the IPD. | **LOW RISK**  ***Were data available for all, or nearly all, participants randomized?***  Data were provided for all randomised participants  ***Other outcomes***  Data for overall recurrence were provided for all randomised participants, data were not provided for local recurrence or metastases | **LOW RISK**  ***Was method of measuring the outcome inappropriate?***  Overall survival was derived from the IPD according to the meta-analysis protocol and SAP.  ***Could measurement of the outcome have differed between intervention groups?***  Checks of the IPD revealed that follow-up of participants was somewhat imbalanced for the few participants remaining alive, but with so few participants in follow-up this is not likely to be problematic.  ***Outcome assessor aware of intervention received?***  This cannot affect the overall survival outcome.  ***Other outcomes***  Data were supplied on ‘any recurrence’ rather than local recurrence or metastases, RFS was derived from IPD. Knowledge of intervention cannot affect the secondary outcomes |
| Trial – Otto | | | |
| LOW RISK  *Was allocation sequence random?*  Randomised using permuted blocks.  Also, IPD checks show that the pattern of allocation is balanced by treatment group over time; there were no major imbalance by group on any day of the week; and there were few weekend randomisations  *Was allocation sequence concealed?*  Central telephone confirmed as method used  *Did baseline differences suggest a problem?*  IPD checks show some imbalance by baseline grade. | **LOW RISK**  ***Were participants aware of their assigned intervention during the trial?***  Blinding not possible in a chemotherapy versus none trial, but awareness cannot affect survival outcome.  ***Were carers and people delivering the interventions aware of participants' assigned intervention during the trial?***  Blinding not possible, but awareness would not affect how treatments were given.  ***Were there deviations from the intended intervention that arose because of the trial context?***  There were no deviations because of the context.  ***Was an appropriate analysis used to estimate the effect of assignment to intervention?*** An intention-to-treat analysis of all randomised participants was derived from the IPD. | **LOW RISK**  ***Were data available for all, or nearly all, participants randomized?***  Data were provided for all randomised participants  ***Other outcomes***  Data were not provided for any recurrence outcomes | **LOW RISK**  ***Was method of measuring the outcome inappropriate?***  Overall survival was derived from the IPD according to the meta-analysis protocol and SAP  ***Could measurement of the outcome have differed between intervention groups?***  Checks of the IPD revealed that follow-up of participants was balanced by treatment group.  ***Outcome assessor aware of intervention received?***  This cannot affect the overall survival outcome.  ***Other outcomes***  Data were not provided for any recurrence outcomes |
| Trial - Sternberg | | | |
| LOW RISK  *Was allocation sequence random?*  Randomised. Minimisation, stratified by institution, pathological T stage and lymph node status.  Also, IPD checks show that the pattern of allocation is steady by treatment group and over time; there were no major imbalance by group on any day of the week; and there were few weekend randomisations.  *Was allocation sequence concealed?*  Randomisation was done centrally at the EORTC headquarters’.  *Did baseline differences suggest a problem?*  IPD checks show well-balanced across baseline characteristics. | **LOW RISK**  ***Were participants aware of their assigned intervention during the trial?***  Blinding not possible in a chemotherapy versus none trial, but awareness cannot affect survival outcome.  ***Were carers and people delivering the interventions aware of participants' assigned intervention during the trial?***  Blinding not possible, but awareness would not affect how treatments were given.  ***Were there deviations from the intended intervention that arose because of the trial context?***  There were no deviations because of the context.  ***Was an appropriate analysis used to estimate the effect of assignment to intervention?*** An intention-to-treat analysis of all randomised participants was derived from the IPD. | **LOW RISK**  ***Were data available for all, or nearly all, participants randomized?***  Data were provided for all randomised participants.  ***Other outcomes***  Local recurrence and metastases data were provided for all participants | **LOW RISK**  ***Was method of measuring the outcome inappropriate?***  Overall survival was derived from the IPD according to the meta-analysis protocol and SAP  ***Could measurement of the outcome have differed between intervention groups?***  Checks of the IPD revealed that follow-up of participants was balanced by treatment group.  ***Outcome assessor aware of intervention received?***  This cannot affect the overall survival outcome.  ***Other outcomes***  All secondary outcomes were derived from IPD. Knowledge of intervention cannot affect the secondary outcomes |
| Trial – Cognetti | | | |
| LOW RISK  *Was allocation sequence random?*  Randomised. Stratified by investigator centre, & nodal involvement.  Also, IPD checks show a slight imbalance by treatment arm, There were no major imbalances by group on any day of the week; and there were few weekend randomisations  *Was allocation sequence concealed?*  Treatment allocated through two coordinating centres using computer-generated lists  *Did baseline differences suggest a problem?*  IPD checks show some imbalance by baseline sex, pTcat, pNcat, grade and PS. | **LOW RISK**  ***Were participants aware of their assigned intervention during the trial?***  Blinding not possible in a chemotherapy versus none trial, but awareness cannot affect survival outcome.  ***Were carers and people delivering the interventions aware of participants' assigned intervention during the trial?***  Blinding not possible, but awareness is unlikely to affect how it these treatments were given.  ***Were there deviations from the intended intervention that arose because of the trial context?***  There were no deviations because of the context.  ***Was an appropriate analysis used to estimate the effect of assignment to intervention?*** An intention-to-treat analysis of all randomised participants was derived from the IPD. | **LOW RISK**  ***Were data available for all, or nearly all, participants randomized?***  Data provided for all randomised participants, including data for 11 participants excluded from original analysis  ***Other outcomes***  Local recurrence and metastases data were provided for all participants | **LOW RISK**  ***Was method of measuring the outcome inappropriate?***  Overall survival was derived from the IPD according to the meta-analysis protocol and SAP.  ***Could measurement of the outcome have differed between intervention groups?***  Checks of the IPD revealed that follow-up of participants was balanced by treatment group.  ***Outcome assessor aware of intervention received?***  This cannot affect the overall survival outcome.  ***Other outcomes***  All secondary outcomes were derived from IPD. Knowledge of intervention cannot affect the secondary outcomes |
| Trial – Stadler | | | |
| LOW RISK  *Was allocation sequence random?*  Randomised. Minimisation used, stratified by age, Stage, Grade and p21 status.  Also, IPD checks show that the pattern of allocation is steady by treatment group and over time; there were no major imbalances by group on any day of the week; and there were no weekend randomisations.  *Was allocation sequence concealed?*  Randomised centrally  *Did baseline differences suggest a problem?*  IPD checks show well-balanced across baseline characteristics. | **LOW RISK**  ***Were participants aware of their assigned intervention during the trial?***  Blinding not possible in a chemotherapy versus none trial, but awareness cannot affect survival outcome.  ***Were carers and people delivering the interventions aware of participants' assigned intervention during the trial?***  Blinding not possible, but awareness is unlikely to affect how it these treatments were given.  ***Were there deviations from the intended intervention that arose because of the trial context?***  There were no deviations because of the context.  ***Was an appropriate analysis used to estimate the effect of assignment to intervention?*** An intention-to-treat analysis of all randomised participants was derived from the IPD. | **LOW RISK**  ***Were data available for all, or nearly all, participants randomized?***  Data were provided for all randomised participants  ***Other outcomes***  Local recurrence and metastases data were provided for all participants | **LOW RISK**  ***Was method of measuring the outcome inappropriate?***  Overall survival was derived from the IPD according to the meta-analysis protocol and SAP.  ***Could measurement of the outcome have differed between intervention groups?***  Checks of the IPD revealed that follow-up of participants was balanced by treatment group.  ***Outcome assessor aware of intervention received?***  This cannot affect the overall survival outcome.  ***Other outcomes***  All secondary outcomes were derived from IPD. Knowledge of intervention cannot affect the secondary outcomes |
| Trial - Zhegalik | | | |
| LOW RISK  *Was allocation sequence random?*  Simple randomisation, performed by a computer software generating random numbers with an equal allocation ratio.  Also, IPD checks show that the pattern of allocation is steady by treatment group and over time; there were no major imbalances by group on any day of the week; and there was one weekend randomisation. Checks highlighted 15 participants with date of randomisation before reported start date of the trial. This was queried with investigator, who explained this was mistake in the manuscript  *Was allocation sequence concealed?*  Randomised centrally  *Did baseline differences suggest a problem?*  IPD checks show some imbalance by baseline sex, pTcat, and grade | **LOW RISK**  ***Were participants aware of their assigned intervention during the trial?***  Blinding not possible in a chemotherapy versus none trial, but awareness cannot affect survival outcome.  ***Were carers and people delivering the interventions aware of participants' assigned intervention during the trial?***  Blinding not possible, but awareness is unlikely to affect how it these treatments were given.  ***Were there deviations from the intended intervention that arose because of the trial context?***  There were no deviations because of the context.  ***Was an appropriate analysis used to estimate the effect of assignment to intervention?*** An intention-to-treat analysis of all randomised participants was derived from the IPD. | **LOW RISK**  ***Were data available for all, or nearly all, participants randomized?***  Data were provided for all randomised participants  ***Other outcomes***  Local recurrence data provided for 85/100 participants and metastases data were provided for 86/100 participants | **LOW RISK**  ***Was method of measuring the outcome inappropriate?***  Overall survival was derived from the IPD according to the meta-analysis protocol and SAP. This was the same definition as in the trial  ***Could measurement of the outcome have differed between intervention groups?***  Checks of the IPD revealed that follow-up of participants was balanced by treatment group.  ***Outcome assessor aware of intervention received?***  This cannot affect the overall survival outcome.  ***Other outcomes***  All secondary outcomes were derived from IPD.. Knowledge of intervention cannot affect the secondary outcomes |

# Supplementary Figure 2: Effect on overall survival by regimen


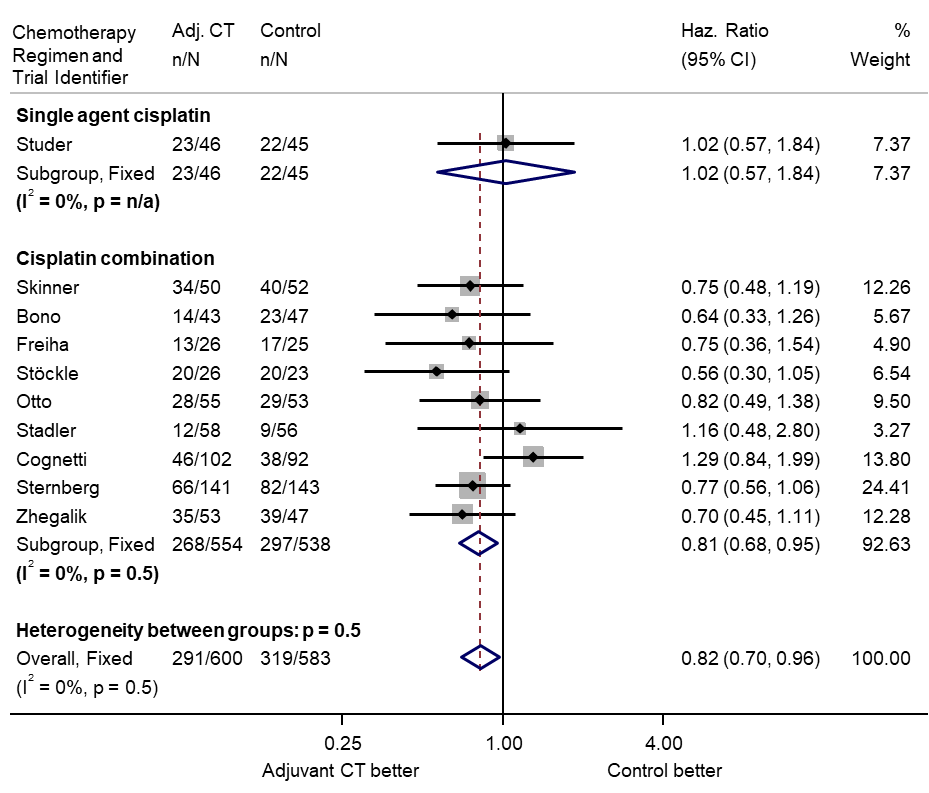


# Supplementary Figure 3: Effect on overall survival by planned (control arm) treatment on relapse

#
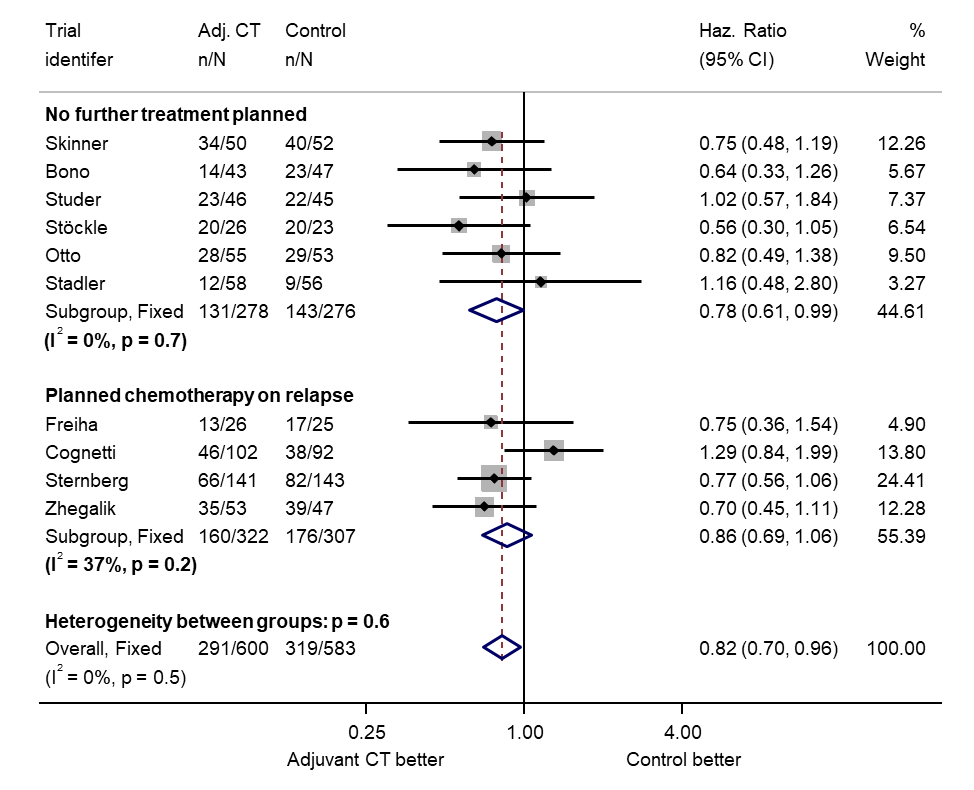


# Supplementary Figure 4: Interactions by trial between the effect of adjuvant chemotherapy on overall survival and pT stage


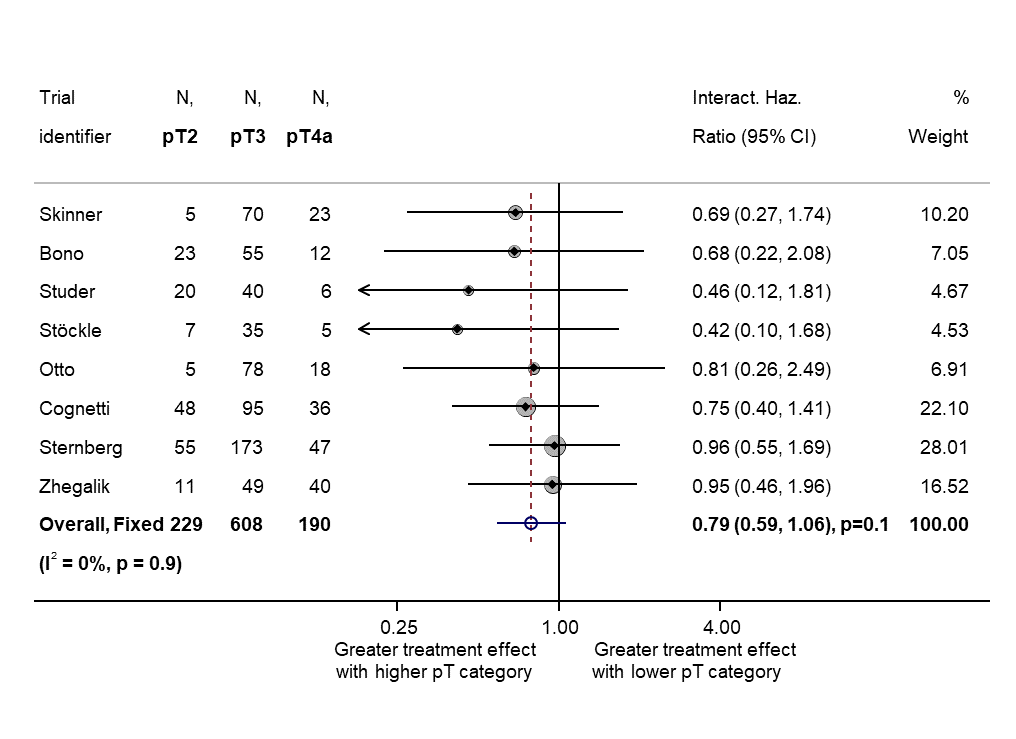


# Supplementary Figure 5: Interactions by trial between the effect of adjuvant chemotherapy on overall survival and pN status


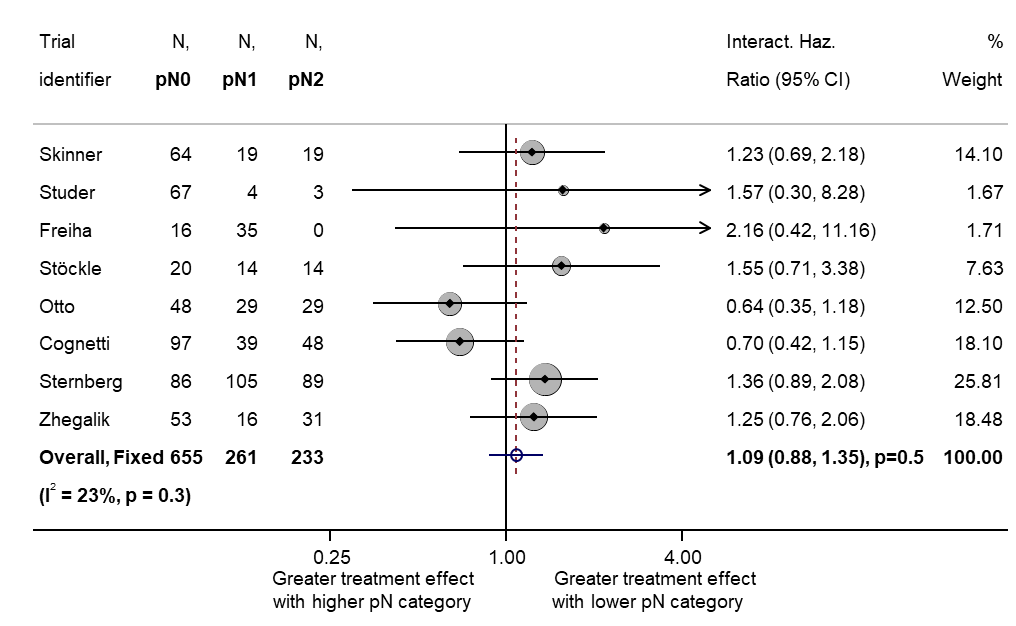


# Supplementary Figure 6: Interactions by trial between the effect of adjuvant chemotherapy on overall survival and p stage (exploratory)


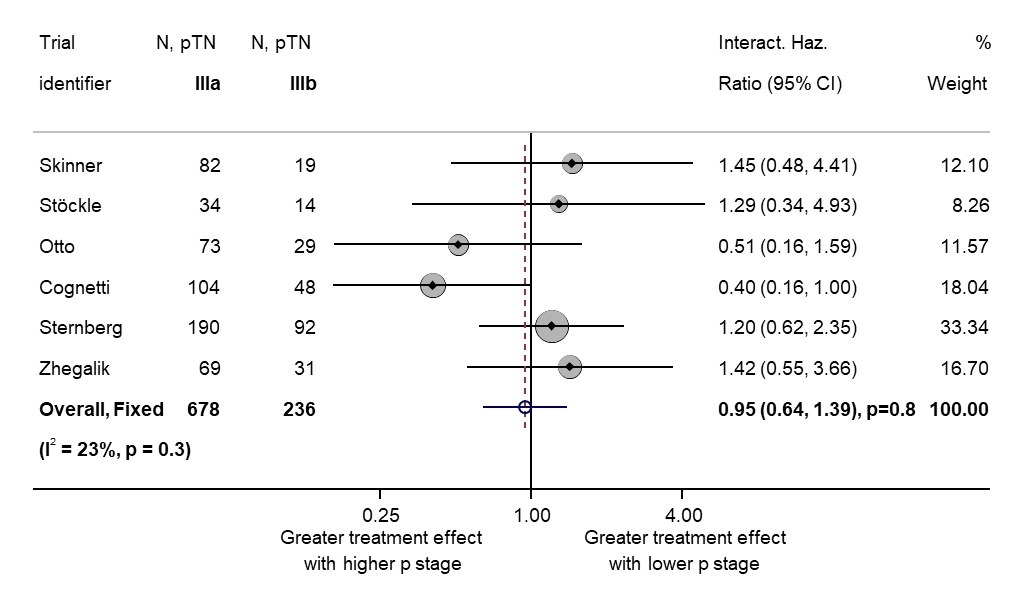


# Supplementary Figure 7: Patterns of locoregional recurrence, metastases and death


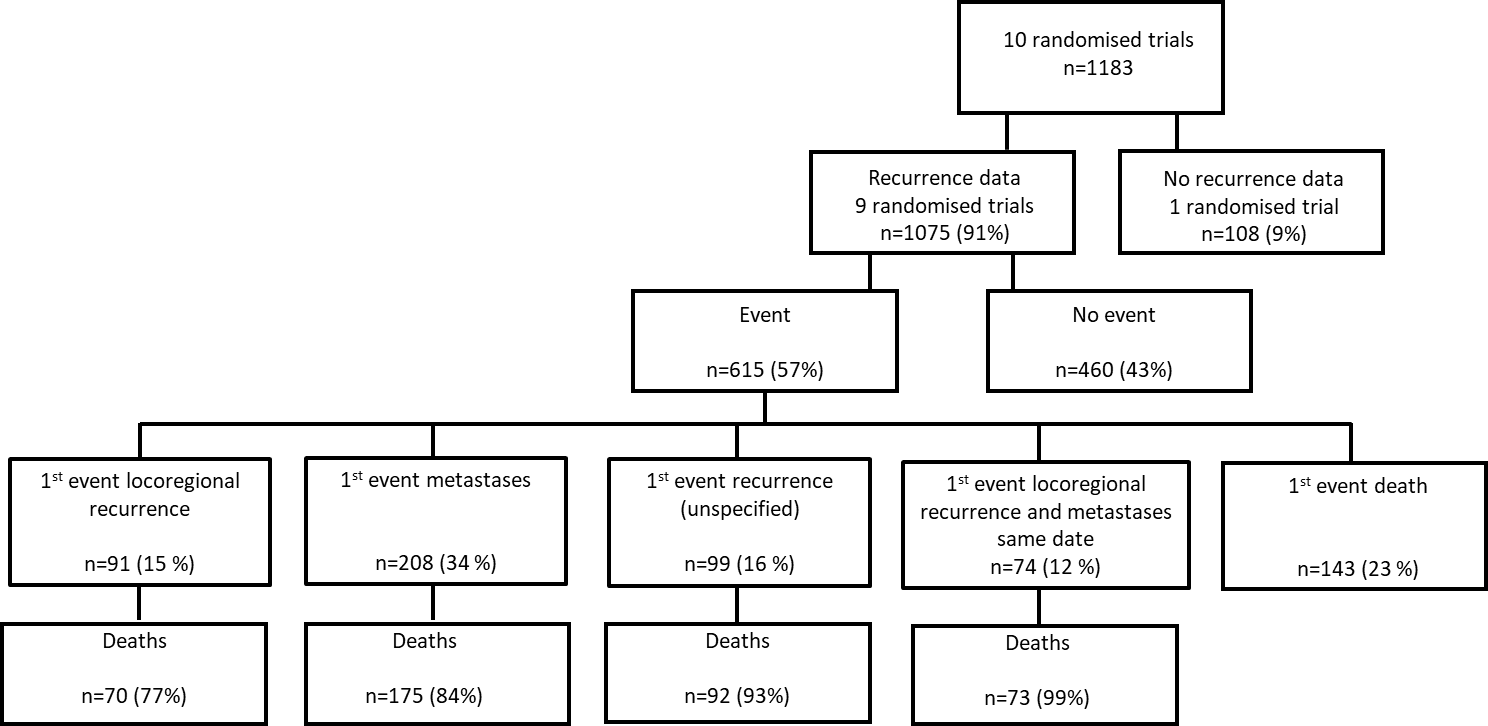


#

# Supplementary material

# Search strategies

**MEDLINE search strategy^[7](#_ENREF_7)^**

1. randomi*ed controlled trial.pt.
2. controlled clinical trial.pt.
3. randomi*ed".ab.
4. placebo.ab.
5. clinical trials as topic.sh.
6. randomly.ab.
7. trial.ti.
8. 1 or 2 or 3 or 4 or 5 or 6 or 7
9. exp animals/ not humans.sh.
10. 8 not 9
11. exp Bladder Neoplasms/
12. (bladder$ adj3 carcinoma$).mp.
13. (bladder$ adj3 canc$).mp.
14. (bladder$ adj3 neoplas$).mp.
15. (bladder$ adj3 tumo?r$).mp.
16. (bladder$ adj3 malignan$).mp.
17. 11 or 12 or 13 or 14 or 15 or 16
18. exp Drug Therapy/
19. (multimodal$ or adjuvant or adjunct$).mp.
20. (together or plus or concurrent or combin$ or add$ or conjuct$).tw.
21. 18 or 19 or 20
22. ((chemotherapy or antineoplastic or anticancer) adj3 agent$).tw.
23. exp chemotherapy adjuvant/
24. exp Antineoplastic agents/
25. 22 or 23 or 24
26. 21 or 25
27. 10 and 17 and 26
28. limit 27 to yr="2005 -Current"

**EMBASE search strategy**

1. randomi*.tw.
2. placebo.mp.

3. double-blind.tw.

4. 1 or 2 or 3

5. exp bladder neoplasms/

6. (bladder$ adj3 carcinoma$).ab,kw,ti.

7. (bladder$ adj3 canc$).ab,kw,ti.

8. (bladder$ adj3 neoplas$).ab,kw,ti.

9. (bladder$ adj3 tumo?r$).ab,kw,ti.

10. (bladder$ adj3 malignan$).ab,kw,ti.

11. 5 or 6 or 7 or 8 or 9 or 10

12. exp drug therapy/

13. (multimodal$ or adjuvant or adjunct$).mp.

14. (together or plus or concurrent or combin$ or add$).mp. or conjuct$.tw.

15.12 or 13 or 14

16. ((chemotherapy or antineoplastic or anticancer) adj3 agent$).tw.

17. exp chemotherapy adjuvant/

18. exp antineoplastic agents/

19. 16 or 17 or 18

20. 15 or 19

21. 4 and 11 and 20

22. limit 21 to yr="2005 - 2017"

**CENTRAL search strategy**

1. MeSH descriptor: [Urinary Bladder Neoplasms] explode all trees

2. blad* near/3 malignan*

3. blad* near/3 canc*

4. blad* near/3 carcinoma*

5. blad* near/3 tumor*

6. blad* near/3 neoplas*

7. #1 or #2 or #3 or #4 or #5 or #6

**ClinicalTrials.gov search strategy**

Condition or disease: Bladder Cancer

Study Type: All studies

Study Results: All Studies

Eligibility (Age): Adult

Additional Criteria (Phase): Phase 3
